# Current Standards of Early Rehabilitation after Anterior Cruciate Ligament Reconstruction in German Speaking Countries—Differentiation Based on Tendon Graft and Concomitant Injuries

**DOI:** 10.3390/ijerph19074060

**Published:** 2022-03-29

**Authors:** Clemens Memmel, Werner Krutsch, Dominik Szymski, Christian Pfeifer, Leopold Henssler, Borys Frankewycz, Peter Angele, Volker Alt, Matthias Koch

**Affiliations:** 1 Department of Paediatric Surgery and Orthopaedics, Clinic St. Hedwig, Barmherzige Brueder Regensburg, KUNO Paediatric University Medical Centre and FIFA Medical Centre of Excellence, University Medical Centre Regensburg, 93053 Regensburg, Germany; 2 Department of Trauma Surgery and FIFA Medical Centre of Excellence, University Medical Centre Regensburg, 93053 Regensburg, Germany; werner.krutsch@ukr.de (W.K.); dominik.szymski@me.com (D.S.); christian.pfeifer@ukr.de (C.P.); leopold.henssler@ukr.de (L.H.); borys.frankewycz@ukr.de (B.F.); peter.angele@ukr.de (P.A.); volker.alt@ukr.de (V.A.); matthias.koch@ukr.de (M.K.); 3 SportDocs Franken, 90455 Nuremberg, Germany; 4 Department of Trauma Surgery, InnKlinikum Altoetting, 84503 Altoetting, Germany; 5 Department of Orthopaedic Surgery and Traumatology, Kantonsspital Baselland, Bruderholz, 4101 Basel, Switzerland

**Keywords:** anterior cruciate ligament reconstruction, early rehabilitation, hamstring tendon graft, bone tendon–bone graft, patella tendon graft

## Abstract

***Background***: Although anterior cruciate ligament reconstruction (ACLR) is a well-established procedure and is standardly performed by orthopedic surgeons all over the world, there does not seem to be a standard protocol for early rehabilitation. The purpose of this study was to give answers to the following questions: (i) Does (a) the use of a specific tendon graft, and (b) potentially additional therapy of concomitant pathologies influence surgeons’ choice of a distinct postoperative rehabilitation protocol after ACLR? (ii) To what extent do these rehabilitation recommendations differ? ***Methods***: Retrospective analysis of currently used early rehabilitation protocols after ACLR in German-speaking countries (GER, AUT and SUI) was conducted. Rehabilitation criteria included weight bearing, range of motion (ROM), the utilization of braces, continuous passive/active motion therapy (CPM/CAM), rehabilitation training and sport-specific training. Tendon grafts were differentiated as hamstring (HAM) and bone–patellar tendon–bone grafts (BTB). Concomitant pathologies included meniscus injuries (+M) and unhappy triad injuries (+UTI). ***Results***: Most of the surveyed protocols were differentiated according to the used tendon graft or additional therapy of concomitant injuries (ACLR-differentiated, *n* = 147 vs. ACLR without graft differentiation, *n* = 58). When comparing ACLR-HAM and ACLR-BTB, significant differences were found regarding weight bearing (*p* = 0.01), ROM (*p* = 0.05) and the utilization of braces (*p* = 0.03). Regarding ACLR+M, an overall significant decelerated rehabilitation could be detected. After ACLR+UTI-therapy, a significant delayed start to full weight bearing (*p* = 0.002) and ROM (*p* < 0.001) was found. ***Conclusions***: Most orthopedic surgeons from German-speaking countries differentiate early rehabilitation after ACLR according to the tendon graft used and therapy of concomitant pathologies. No consensus about early rehabilitation after ACLR is available. However, tendencies for an accelerated rehabilitation after ACLR-BTB and a more restrained rehabilitation of multiple injured knees were detected.

## 1. Introduction

Injuries of the anterior cruciate ligament (ACL) are among the most common ligamentous injuries of the knee joint [1,2,3,4,5]. Recommendations for therapy depend on the athletic demands, as well as muscular compensatory potential [6,7]. Surgical therapy is especially indicated for young, active patients, as well as for patients with concomitant pathologies and persistent instability of the knee joint [8,9]. However, there are many techniques for ACL reconstructive surgery (ACLR), especially regarding the available tendon grafts, such as hamstring tendons (HAM) or bone–patellar tendon–bone grafts (BTB). Advantages and disadvantages concerning the different tendon grafts have been widely discussed in various clinical and biomechanical studies [6,7,10,11,12]. Besides the type of surgical technique, the overall outcome of ACLR depends significantly on the type and intensity of rehabilitation [13,14,15]. The early rehabilitation phase in particular is crucial for the outcome after ACLR, as the healing of the graft and possibly concomitant lesions of the meniscus or collateral ligaments starts at this point in time. During the early rehabilitation period, the restoration of the postoperative range of motion (ROM), restrengthening of muscles and neuromuscular training is essential [14,15,16]. Furthermore, there is significant data about a correlating effect between the early rehabilitation phase and postoperative complications, such as arthrofibrosis and infection rate [3,17]. For example, failing to achieve full active and passive ROM within the first weeks after surgery can affect long-term outcome measures such as pain, gait and function, which can in turn lead to arthrofibrosis. The early rehabilitation phase with all its categories thus paves the way for a best possible and successful postoperative outcome.

Looking at orthopedics worldwide, there are numerous national and international associations and research groups that provide post-treatment recommendations after ACLR, which are updated regularly, e.g., by MOON (Multicenter Orthopaedic Outcomes Network, USA), KNGF (Royal Dutch Society for Physical Therapy, Amersfoort, The Netherlands), the DOA (Dutch Orthopaedics Association, Hertogenbosch, The Netherlands) or the DGOU (German Association of Orthopaedics and Trauma Surgery, Berlin, Germany). However, they differ not only in terms of content concerning the recommendations, but also in terms of quality and applicability to clinical practice. What is striking here is that most of them do not address different graft types or concomitant injuries [18,19,20,21,22], such as meniscus injuries or meniscus and medial collateral ligament injuries in terms of unhappy triad injuries (UTIs) [23,24,25]. It is also not known how orthopedic surgeons currently fill this gap of evidence in clinical practice and what their rehabilitation recommendations for physiotherapists and patients are.

Therefore, the research questions for this study were:

(1) Does (a) the use of a specific tendon graft, and (b) potentially additional therapy of concomitant pathologies influence surgeons’ choice of a distinct postoperative rehabilitation protocol after ACLR?
(2) To what extent do these rehabilitation recommendations differ?

## 2. Materials and Methods

*Design*—The medical device company *OPED* (GmbH, Valley, Germany) developed a tabular template for creating a post-treatment protocol after ACLR, which *OPED* made freely available to orthopedic departments and outpatient orthopedic centers in German-speaking countries, so that the associated orthopedic surgeons could use the template to create their individual post-treatment rehabilitation standard using the categories described in Table 1. *OPED* itself was not involved in the content of the rehabilitation protocols. All protocols were collected and blinded for study purposes. The investigated protocols contain general early rehabilitation recommendations of orthopedic surgeons given to their patients and physiotherapists. The investigation of these protocols focused on the described use of different tendon grafts, such as HAM grafts or BTB grafts, as well as the presence and additional therapy of concomitant injuries such as meniscus lesions or UTIs and the categories of early rehabilitation as described in Table 1 and Table 2.

*Participants*—Rehabilitation protocols for early rehabilitation after ACLR in written form (*n* = 205), which are currently used in daily routines in German-speaking countries (GER, AUT and SUI), were surveyed for this qualitative study. These originated from 120 different orthopedic institutions, of which 63 were outpatient centers and 57 were clinical centers, 4 of which in turn were university medical centers.

*Measures*—The included early rehabilitation protocols were analyzed regarding the surgery performed, particularly the type of tendon graft used, the therapy of concomitant pathologies and the following rehabilitation categories (see Table 1 and Table 2). The early rehabilitation categories included postoperative weight-bearing recommendations, restriction of range of motion (ROM), the utilization of braces, recommended continuous passive/active motion (CPM/CAM), as well as the recommended start of rehabilitation training and specific training. Rehabilitation training was defined as any kind of basic sport activity for the restoration of coordination and muscle strengthening, such as the use of an ergometer, cycling, aqua jogging, general strength training or crawling. Specific training included roadwork, coordination and proprioception training, as well as sport-specific training. Criteria for the rehabilitation progress, such as the period and time points of limitations and recommendations, were registered. All evaluated categories, excluding rehabilitation or specific training, were analyzed for the time periods up to three days postoperative, seven days postoperative and all weekly intervals until full weight bearing and full range of motion were allowed or no brace or no further CPM/CAM training was necessary.

*Data analysis*—Statistical analysis was performed with SPSS^®^ (Version 25, IBM, Armonk, NY, USA). Data are presented as mean ± SD or absolute and relative frequencies. Continuous data between two or more groups were compared by an analysis of variance (ANOVA). Categorical data were compared by using the chi-squared test of independence. In case of significance, further post hoc tests (according to Fisher’s least significant difference) were performed. A probability (*p*) value of ≤0.05 was considered to be significant for each test. Graphical illustrations were generated with GraphPad Prism^®^ (Version 5.01, GraphPad Software, La Jolla, CA, USA) and Microsoft PowerPoint 2013^®^ (Microsoft Corporation, Redmond, WA, USA).

## 3. Results

### 3.1. Algorithm of Evaluation

In total, 205 early rehabilitation protocols after ACLR were analyzed. Of these, 57 protocols provided information about early rehabilitation after ACLR using HAM grafts (ACLR-HAM), 21 for BTB grafts (ACLR-BTB), 52 for ACLR with concomitant meniscus repair (ACLR+M) and 17 for UTI (ACLR+UTI). Another 58 protocols were available for ACLR without clear differentiation of the used graft type (ACLR-w/o-gd). The respective evaluation and comparison of the early rehabilitation categories is described below.

### 3.2. Categories of Early Rehabilitation

#### 3.2.1. Weight Bearing

**Figure 1:**
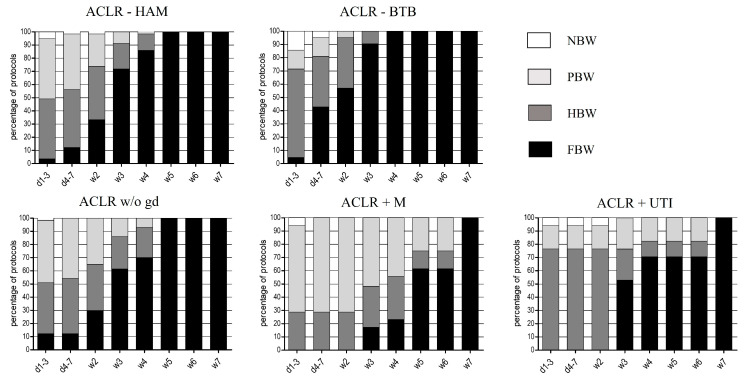
Early rehabilitation phase after ACLR concerning weight bearing, expressed in percentage of protocols (%). NBW: no body weight, PBW: partial body weight, HBW: half body weight, FBW: full body weight, d: day, w: week.

When comparing ACLR-HAM to ACLR-BTB in regard to the recommended regain of weight bearing, loading after ACLR-BTB is allowed significantly earlier (ACLR-HAM: 2.0 ± 1.1 weeks; ACLR-BTB: 1.2 ± 0.7 weeks, *p* = 0.01). The one-week earlier return to full weight bearing (ACLR-HAM after four weeks and ACLR-BTB after three weeks) was found in all post-surgery recommendations (consensus found in 100% of the evaluated rehabilitation protocols, see Figure 1).

When additional therapy of meniscus injuries was performed, the protocols showed a significant delayed increase in load compared to ACLR-w/o-gd of two weeks (*p* < 0.001). When comparing ACLR+UTI to ACLR-w/o-gd, the delay of increase in weight bearing was one week (mean time to full weight bearing: ACLR-w/o-gd: 2.3 ± 1.4 weeks; ACLR+M: 4.0 ± 1.1 weeks, *p* < 0.001; ACLR+UTI: 3.4 ± 1.8 weeks, *p* = 0.002). A two-week delayed return to full weight bearing was found in all post-surgery recommendations (full consensus) (see Figure 1).

#### 3.2.2. Range of Motion (ROM)

**Figure 2:**
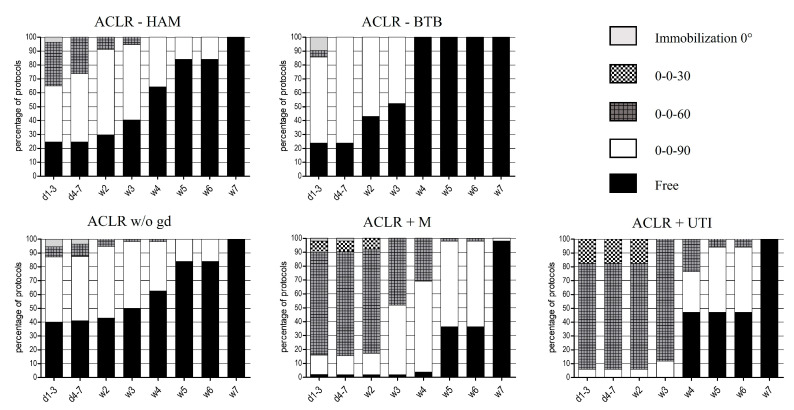
Early rehabilitation phase after ACLR concerning range of motion (ROM), expressed in percentage of protocols (%). d: day, w: week.

The recommended allowance of ROM was found to be twice as fast after ACLR-BTB when compared to ACLR-HAM (100% of protocols, common consensus). The mean time of recommended full ROM after ACLR-BTB was found to be one week earlier compared to ACLR-HAM, which was statistically significant (time to full ROM: ACLR-HAM: 2.7 ± 2.0 weeks; ACLR-BTB: 1.8 ± 1.3 weeks, *p* = 0.050) (see Figure 2).

When comparing isolated ACLR-w/o-gd to ACLR+M or ACLR+UTI, the additional therapy of concomitant pathologies led to a delay in allowed ROM of almost three weeks (time to full ROM: ACLR-w/o-gd: 2.4 ± 2.3 weeks; ACLR+M: 5.2 ± 1.3 weeks, *p* < 0.001; ACLR+UTI: 4.6 ±1.5 weeks, *p* < 0.001) (see Figure 2).

#### 3.2.3. Utilization of Braces

**Figure 3:**
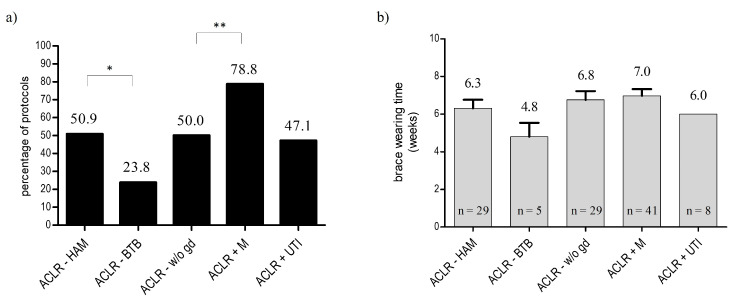
Evaluation of the recommendations regarding utilization of braces after ACLR: (**a**) rate of recommendations, expressed in percentage of protocols (%); (**b**) period of recommended wearing time (* *p* < 0.05, ** *p* < 0.01).

Postoperative external stabilization of the knee joint after ACLR with the use of a brace was recommended in just a quarter of the rehabilitation protocols after ACLR-BTB (*n* = 5; 23.8%). In contrast, almost half of the evaluated concepts after ACLR-HAM recommended the use of braces (*n* = 29; 50.9%; *p* = 0.03). Even if the recommended wearing time of the brace differed by almost two weeks, no significance was detected when comparing ACLR-BTB (4.8 weeks ± 1.6 weeks) to ACLR-HAM (6.3 ± 2.5 weeks; *p* = 0.2) (see Figure 3a,b).

Regarding ACLR with the additional treatment of concomitant injuries, the use of braces was recommended, particularly after ACLR+M (*n* = 41; 79% of the evaluated ACRL+M protocols). In contrast, just half of the rehabilitation concepts utilized braces after ACLR-w/o-gd (*n* = 29; 50%) and ACLR+UTI (*n* = 8; 47%). Analyzing the brace-wearing time, the mean recommended period ranged from six to seven weeks (ACLR-w/o-gd: 6.8 ± 2.5 weeks; ACLR+M: 7.0 ± 2.2 weeks; *p* = 0.69; ACLR+UTI: 6.0 ± 0.0 weeks; *p* = 0.39) (see Figure 3a,b).

#### 3.2.4. Continuous Passive/Action Motion Therapy (CPM/CAM)

**Figure 4:**
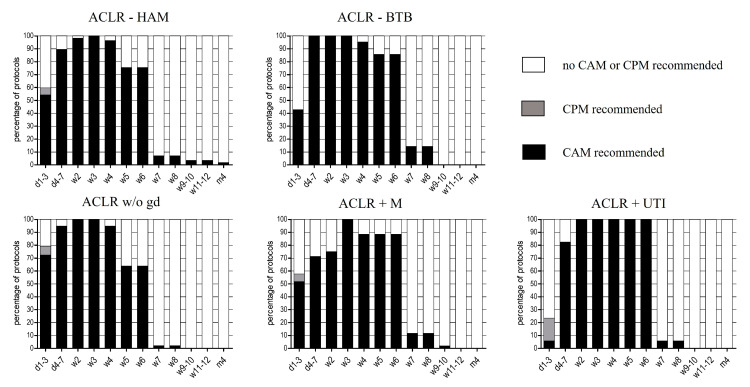
Early rehabilitation phase after ACLR concerning recommendations of continuous passive/active motion (CPM/CAM), expressed in percentage of protocols (%). d: day, w: week, m: month.

When comparing ACLR-HAM and ACLR-BTB, CPM was only recommended in a few protocols after ACLR-HAM for the first three postoperative days. CAM was recommended in a high percentage of all evaluated rehabilitation algorithms from the fourth postsurgical day on. A common consensus was found after one week, where all institutions recommended CAM (see Figure 4). Differences between the treatment groups were not significant. Regarding the period of CAM therapy for both types of tendon graft, a mean of six weeks was recommended (period of CAM therapy: ACLR+HAM: 5.8 ± 1.9 weeks; ACLR+BTB: 6.0 ± 1.2 weeks, *p* = 0.71). After ACLR, in combination with therapy of meniscus injuries or UTIs, just a few protocols recommended the use of CPM for the first three postoperative days. In contrast, CAM therapy was strongly recommended. Recommendation for CAM was limited to six postoperative weeks (see Figure 4).

#### 3.2.5. Start of Rehabilitation and Specific Training

**Figure 5:**
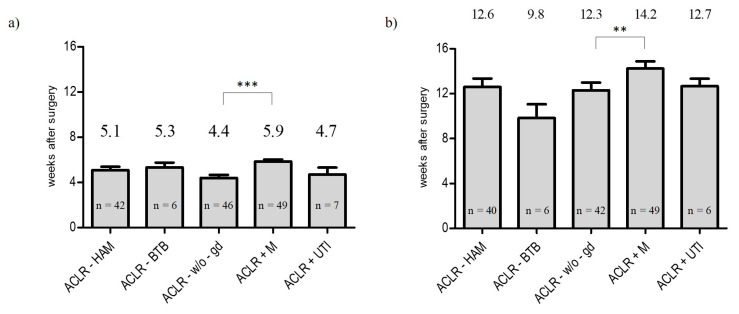
Evaluation of the recommended start of (**a**) rehabilitation training and (**b**) specific training after ACLR (** *p* < 0.01, *** *p* < 0.001).

In ACLR-HAM and ACLR-BTB, rehabilitation training was recommended to start after a mean of almost five weeks without significant difference (start rehabilitation training: ACLR-HAM: 5.1 ± 1.9 weeks; ACLR-BTB: 5.3 ± 1.0 weeks, *p* = 0.74) (see Figure 5a). In the case of treatment of a concomitant injury, significant differences were detected in comparison to ACLR-w/o-gd. After additional meniscus repair, rehabilitation training was recommended to start after an average of six weeks (5.9 ± 1.3, *p* < 0.001). In contrast, rehabilitation training after ACLR-w/o-gd and ACLR+UTI was recommended to start after 4.5 weeks (ACLR-w/o-gd: 4.4 ± 1.7 weeks; ACLR+UTI: 4.7 ± 1.7 weeks, *p* = 0.65) (see Figure 5a). The start of specific training after ACLR-BTB was recommended 2.5 weeks earlier in comparison to the ACLR-HAM group (ACLR-HAM: 12.6 ± 4.7 weeks; ACLR-BTB: 9.8 ± 3.0 weeks, *p* = 0.17). The recommended start of specific training after ACLR+M was delayed by almost two weeks (14.2 ± 4.5 weeks) in comparison to ACLR-w/o-gd (12 ± 4.4 weeks, *p* = 0.036) and ACRL+UTI (12.7 ± 1.6 weeks; *p* = 0.85) (see Figure 5b).

## 4. Discussion

The main finding of this study is that almost three-quarters of the evaluated orthopedic recommendations differ in the early rehabilitation of ACLR in regard to different graft types and therapy of concomitant pathologies. This is an important fact and shows an awareness of orthopedic surgeons of the requirement of individualized rehabilitation concepts to achieve optimal clinical outcomes and return to sports and work. This finding is of particular interest, since current national and international guidelines for rehabilitation after ACLR only provide a basic rehabilitation description, without connecting it to different graft choices or adaptations due to the additional therapy of concomitant injuries [19,22,26]. In the case of concomitant injuries, the particular pathologies and complaints of the additional lesions require special attention, especially in the early rehabilitation period. Thus, the specification of rehabilitation concepts increases the quality of care for patients but complicates the development of consistent rehabilitation algorithms.

In the analyzed early rehabilitation protocols, no general consistent recommendations were found in regard to weight bearing, ROM or the utilization of braces after ACLR and possible additional treatment of concomitant pathologies. Similarly, wide disparities have already been found in a survey of members of the “American Orthopedic Society of Sports Medicine” and the “Arthroscopy Association of North America” [27]. However, there are clear trends that respect the individual character of each kind of therapy. When looking into the two solely graft-replacing techniques (ACLR-HAM and ACLR-BTB), an accelerated early rehabilitation pattern was found in ACLR-BTB. Here, a significantly earlier allowance was found in the recommendations for weight bearing, ROM and the utilization of braces. The early increase in loading and ROM, as well as the low recommendation rate for the use of braces and the reduced period of wearing a brace, might be explained by the higher initial stability of BTB grafts caused by the bone-to-bone contact in the femoral and tibial tunnel which are accompanied by a good healing character [6,28]. Regarding the return to mobility and physical fitness, almost consistent recommendations were given. In contrast, the current literature recommends no extended weight-bearing limitation for both types of tendon graft. In several studies, no negative effects by immediate full weight bearing were detected [21,23,29,30]. Rather, early weight bearing seemed to improve the quadriceps muscle function and reduce the risk for patellofemoral pain [21,23,29,30], which is particularly associated with the choice of BTB grafts [6,10]. However, the DGOU tend to delay full weight bearing after ACLR for two weeks, similar to the survey’s results (ACLR-HAM: 2.0 ± 1.1 weeks; ACLR-BTB: 1.2 ± 0.7 weeks) [19,31], whereas the MOON Guidelines recommend immediate weight bearing after ACLR and so does the KNGF, as long as the gait pattern is correct [21,22]. Analogously, many studies showed that the postoperative use of braces after ACLR was not beneficial for the outcome nor for early rehabilitation [13,22,32,33]. However, the more restrictive recommendations of the increase in ROM after ACLR-HAM might be explained by the weakening of the knee flexors due to the harvesting of HAM tendons, which are essential for joint stability in a flexed knee position [6,34]. Harvesting BTB grafts out of the extensor chain is associated with a minor impairment of the knee flexors; thus, there is no urgent need for the restriction of knee flexion [35].

In the case of ACLR+M and ACLR+UTI, a significantly decelerated start and increase in weight bearing, as well as ROM, was recommended compared to isolated ACLR. Although the principles of ACL healing are the same, orthopedic surgeons acknowledge the additional injuries of the meniscus and collateral ligament as a potential multiple trauma and therefore assume longer healing times. As a consequence, prolonged periods of partial weight bearing and prolonged ROM limitations are implemented. However, there is no evident data in the current literature concerning advantages for accelerated or conservative rehabilitation strategies after meniscus repair [36,37]. In order to safeguard the healing process, additional external stabilization with a brace was recommended, particularly after ACLR+M but not after ACLR+UTI. However, the overall recommended time of brace usage did not differ significantly. Limited literature supports the recommendation of using braces after isolated meniscus repair, as well as medial collateral ligament injuries, to reduce stress on the injured structures [38,39]. Rather, early mobilization is preferred. In the evaluated protocols, there is a consensus about early physiotherapy-guided mobilization, initially by CAM and followed by rehabilitation training and specific training. Additionally, in the currently available literature, an early start of rehabilitation seems to have a beneficial effect on the outcome. Rosso et al. showed that starting physiotherapy after ACLR greater than one month postoperatively is associated with a poorer outcome [40]. Furthermore, many studies suggest that a longer period of rehabilitation training of up to 12 months could improve the rate of return to sport and work [21,30,40,41].

Certainly, the present study has some limitations. Due to the retrospective design, the results lack factors such as individual modification of the rehabilitation protocol by orthopedics, clinical outcome, the patients’ compliance or sociodemographic data such as gender or age. Furthermore, technical details such as meniscus lesion patterns and the kind of meniscus repair, as well as therapeutic information concerning the therapy of medial collateral ligament injuries in context to UTI, are not available. Rehabilitation data about concomitant cartilage injuries are also missing, as they were not available in the protocols. The evaluated rehabilitation protocols cover the early period of rehabilitation up to 12 weeks post-surgery, while regeneration and return to work/sport require a longer period of rehabilitation and are therefore not covered by this data sample. However, this study reveals the clinical routine and daily practices of today’s orthopedic health care providers and therefore shows the current state of play in the postoperative care of ACL-related knee injuries.

## 5. Conclusions

In summary, this study presents detailed information about the status quo of currently used early rehabilitation concepts after ACLR for the first time. Almost three-quarters of orthopedic surgeons in German-speaking countries implement into their postoperative care protocols the choice of a tendon graft and the treatment of concomitant injuries. Even if no consistent rehabilitation algorithms are available, there are trends respecting the individual pathology and consecutive therapy. However, further prospective research is required to investigate the evidence of the currently evaluated rehabilitation concepts and to create a guideline outlining all different aspects of ACLR and the treatment of concomitant injuries.

## Figures and Tables

**Table 1:** Categories of early rehabilitation.

Categories of Early Rehabilitation	
Weight bearing	no body weight (NBW)
	partial body weight (PBW; loading up to 20 kg)
	half body weight (HBW; loading more than 20 kg)
	full body weight (FBW; unlimited loading)
Range of motion (extension/flexion)	immobilization 0°
	0-0-30°
	0-0-60°
	0-0-90°
	free
Utilization of braces	yes/no
	recommended wearing time (weeks)
Continuous passive/active motion	no CPM/CAM recommended
	CPM recommended
	CAM recommended
Start of rehabilitation training	weeks after surgery
Start of specific training	weeks after surgery

**Table 2:** Evaluated groups differentiated according to the used tendon graft +/− additional therapy of concomitant injuries.

ACLR—Groups	
ACLR—HAM	ACLR using hamstring tendon grafts
ACLR—BTB	ACLR using bone–patellar tendon–bone grafts
ACLR—w/o gd	ACLR without graft differentiation
ACLR+M	ACLR with additional meniscus repair
ACLR+UTI	ACLR with additional meniscus and medial collateral ligament therapy

## Data Availability

The data presented in this study are available on request from the corresponding author.
